# Utility of plasma anti-gSG6-P1 IgG levels in determining changes in *Anopheles gambiae* bite rates in a rural area of Cameroon

**DOI:** 10.1038/s41598-024-58337-8

**Published:** 2024-06-21

**Authors:** Glwadys Cheteug Nguetsa, Emmanuel Elanga-Ndille, Estelle Géraldine Essangui Same, Tatiana Nganso Keptchouang, Stanilas Elysée Mandeng, Wolfgang Ekoko Eyisap, Jérome Achille Binyang, Balotin Fogang, Lynda Nouage, Micheal Piameu, Lawrence Ayong, Josiane Etang, Samuel Wanji, Carole Else Eboumbou Moukoko

**Affiliations:** 1 Malaria Research Unit, Centre Pasteur Cameroon, P.O. Box 1274, Yaoundé, Cameroon; 2 Department of Microbiology and Parasitology, Faculty of Sciences, The University of Buea, P.O. Box 63, Buea, Cameroon; 3 Department of Animal Biology, Faculty of Sciences, The University of Dschang, P.O. Box 96, Dschang, Cameroon; 4 Department of Medical Entomology, Centre for Research in Infectious Diseases, P.O. Box 13591, Yaoundé, Cameroon; 5 Department of Biological Sciences, Faculty of Medicine and Pharmaceutical Sciences, The University of Douala, P.O. Box 2701, Douala, Cameroon; 6 Department of Animal Biology and Physiology, Faculty of Sciences, The University of Yaoundé, P.O. Box 337, Yaounde 1, Cameroon; 7 Laboratoire de Recherche sur le Paludisme, Organisation de Coordination pour la lutte Contre les Endémies en Afrique Centrale (OCEAC), P.O. Box 288, Yaoundé, Cameroon; 8 Ecole des Sciences de La Santé, Université Catholique d’Afrique Centrale, P.O. Box 1110, Yaoundé, Cameroon; 9 Laboratory of Parasitology, Mycology and Virology, Postgraduate Training Unit for Health Sciences, Postgraduate School for Pure and Applied Sciences, The University of Douala, P.O. Box 2701, Douala, Cameroon

**Keywords:** Anti-gSG6-P1 IgG response, Seasonal transmission, Mosquito density, Human biting rate, Mosquito infection rates, Rural area, Cameroon, Immunology, Biomarkers, Diseases

## Abstract

The applicability of the specific human IgG antibody response to *Anopheles gambiae* salivary Gland Protein-6 peptide 1 (gSG6-P1 salivary peptide) as a biomarker able to distinguish the level of exposure to mosquito bites according to seasonal variations has not yet been evaluated in Central African regions. The study aimed to provide the first reliable data on the IgG anti-gSG6-P1 response in rural area in Cameroon according to the dry- and rainy-season. Between May and December 2020, dry blood samples were collected from people living in the Bankeng village in the forest area of the Centre region of Cameroon. Malaria infection was determined by thick-blood smear microscopy and multiplex PCR. The level of IgG anti-gSG6-P1 response, was assessed by enzyme-linked immunosorbent assay. Anopheles density and aggressiveness were assessed using human landing catches. The prevalence of malaria infection remains significantly higher in the rainy season than in the dry season (77.57% vs 61.44%; p = 0.0001). The specific anti-gSG6-P1 IgG response could be detected in individuals exposed to few mosquito bites and showed inter-individual heterogeneity even when living in the same exposure area. In both seasons, the level of anti-gSG6-P1 IgG response was not significantly different between *Plasmodium* infected and non-infected individuals. Mosquito bites were more aggressive in the rainy season compared to the dry season (human biting rate-HBR of 15.05 b/p/n *vs* 1.5 b/p/n) where mosquito density was very low. Infected mosquitoes were found only during the rainy season (sporozoite rate = 10.63% and entomological inoculation rate-EIR = 1.42 ib/p/n). The level of IgG anti-gSG6-P1 response was significantly higher in the rainy season and correlated with HBR (p ˂ 0.0001). This study highlights the high heterogeneity of individual’s exposure to the *Anopheles gambiae s.l* vector bites depending on the transmission season in the same area. These findings reinforce the usefulness of the anti-gSG6-P1 IgG response as an accurate immunological biomarker for detecting individual exposure to *Anopheles gambiae s.l.* bites during the low risk period of malaria transmission in rural areas and for the differentiating the level of exposure to mosquitoes.

## Introduction

Vector-borne diseases are the most important illnessin terms of human health burden, both with regard to morbidity and mortality, accounting for 17% of all infectious sickness^1^. Malaria infection is transmitted by the bites of infected female *Anopheles* (*An*.) mosquitoes and remains the most common parasitic disease in low-income countries, although significant progress has been made in reducing the global malaria burden^2^. In Cameroon, nearly 7 million malaria cases and 15,048 of deaths were reported in 2020^2^.

In sub-Saharan Africa, the risk of malaria is traditionally considered to be significantly higher in rural areas^3,4^ and this could be explained by environmental factors that play a key role in the mosquito density and consequently in the malaria transmission^5^. Mosquitoes find favourable breeding grounds in pools of clean stagnant water, usually in swamps, puddles, ponds, rice fields^6,7^. Other factors such as climate, season or poorly constructed houses with bushes around them also contribute to a higher risk of malaria infection^8^.

Most of the major control strategies have been widely implemented in the rural areas. Two forms of vector control (insecticide-treated mosquito nets (ITNs) and indoor residual spraying of insecticides) are effective in a wide range of circumstances^9^. In Cameroon, the mass distribution campaign (MDC) of long-lasting insecticidal nets (LLINs) which is the principal vector control strategy against mosquito’s *bites* was implemented in 2011, with more than 8.5 million of LLINs distributed throughout the country^10^. This was followed by a second mass distribution in 2015 and a third with the distribution of about eight million LLINs in 2019^11^. However, little is known about the impact of these strategies regarding at the individual level of exposure to mosquitoes bites. Assessing human exposure to Anopheles vectors, and therefore the risk of malaria transmission, helps to evaluate the effectiveness of vector control interventions. Currently, the assessment of the human exposure to the vector bites is based on entomological methods that estimate the entomological inoculation rate (EIR), which is the standard method and is usually interpreted as the number of infectious bites received by an individual per unit of time^3,12^. However, this parameter has significant drawbacks and limitations, such as inaccuracy due to the micro-heterogeneity of malaria transmission, especially in areas of low transmission areas, during the dry season, urban areas and at high altitudes locations^12–14^. It is also expensive, time-consuming, not suitable for use at the individual level, and sometimes very difficult or impossible, especially when mosquito densities are very low^15^. EIR also depends on both human biting rate (HBR) and mosquito infection rate (IR), which can be difficult to estimate in low transmission settings and may lack sensitivity because the number of *Plasmodium*-positive samples is insufficient to estimate the sporozoite rates (SR) and the number of mosquitoes collected is often very low in the dry season^16,17^. In addition, human-landing catches (HLCs), which are commonly used to collect adult mosquitoes for human exposure assessment, have limited feasibility due to ethical considerations and logistical constraints, and results cannot be extrapolated to children^18–20^.

In this context, an alternative serological method was developed to measure human exposure to arthropods bites^21,22^ and the specific immunoglobulin G (IgG) response to the gSG6-P1 peptide was validated as a biomarker of human exposure to *Anopheles* bites^23^. This IgG response to gSG6-P1 can be used in the context of low exposure to *Anopheles* bites and in local transmission hotspots to assess malaria risk in urban and rural areas. This tool was also shown to be useful to assess the effectiveness vector control interventions in different settings, including West Africa (Ivory Coast, Senegal), East Africa (Angola)^24–27^, the Americas^28,29^ and Asia^30,31^. Some studies have also shown that this immune response correlates with the *Plasmodium* infection in humans^26,29,32,33^. In addition, studies have shown that specific IgG responses to this gSG6-P1 peptide provide an accurate assessment of low and very low levels of exposure to the major malaria vector in Africa, *An*. *gambiae,* as well as to the second major malaria vector in West Africa, *An*. *funestus*^23,34^.

Recently, for the first time in the Central African region, we conducted a study in Cameroon that validated the use of gSG6-P1 IgG response as a biomarker of exposure to malaria vector bites in mainland and island areas of the Littoral region, where *An. gambiae s.l.* is the main vector^35^. More interestingly, we recently reported in Southern Cameroon that IgG response to *An. gambiae* gSG6-P1 salivary peptide could be detected in humans exposed mainly to *An. moucheti* and *An. paludis* bites, known as secondary malaria vectors in Cameroon^36^. Based on this and all previous studies conducted from the Central African region, the present prospective population-based study aims to quantify the level of anti-gSG6-P1 salivary peptide IgG response according to the density of *Anopheles* mosquitoes and seasonal variation (rainy- and dry-season parasite transmission) in a village of the Centre region of Cameroon.

## Methods

### Study design and population

**Figure 1:**
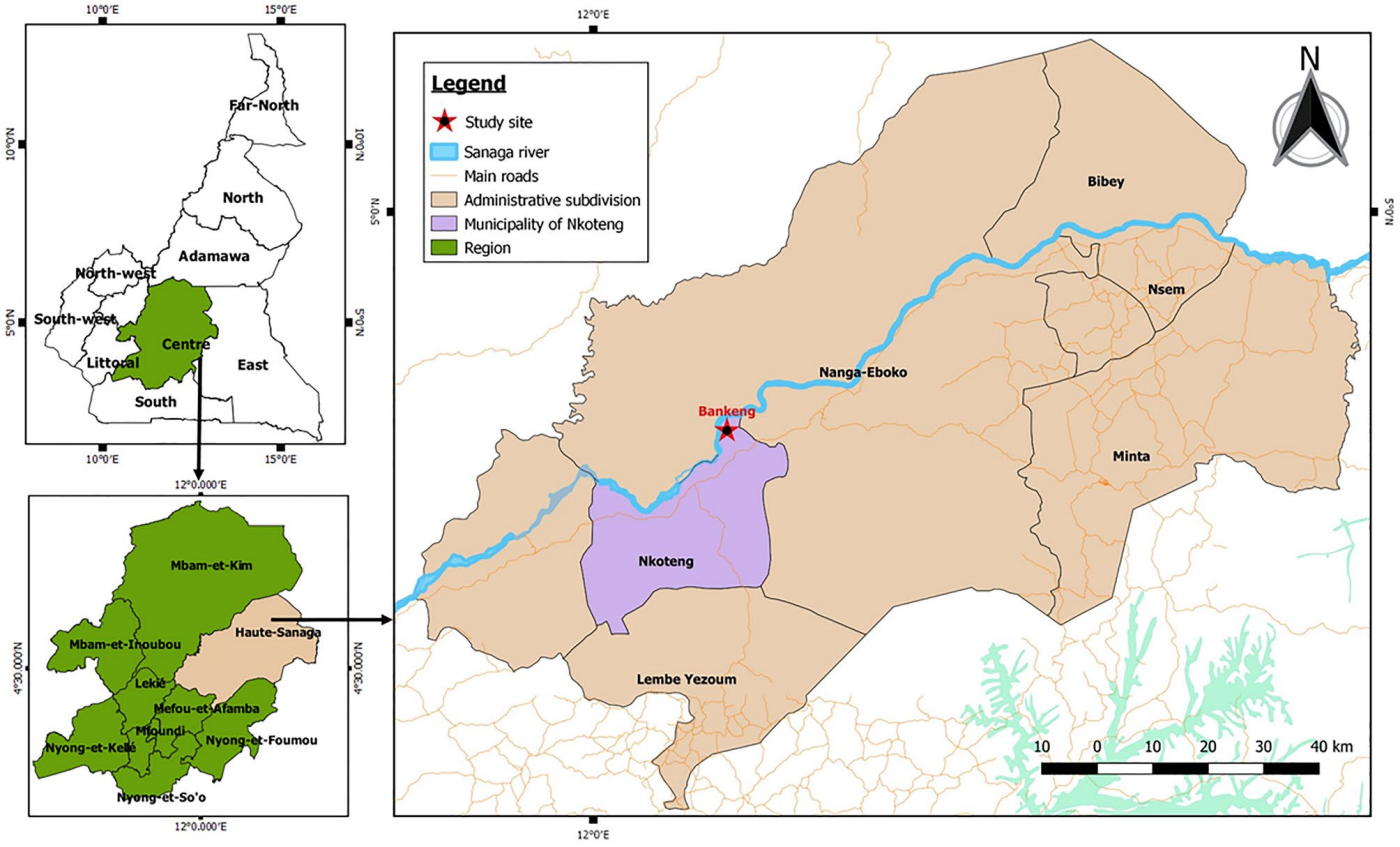
Map showing the location of the Bankeng, a small Village in Central Cameroon. Map was created using QGIS software v2.18 (https://docs.qgis.org/2.18/en/docs/index.html).

A cross-sectional community-based study was conducted in the Bankeng village (4° 38,043″ N; 12° 13,003″ E), located in the Department of “Haute Sanaga”, Central Region of Cameroon, during the rainy season (May 2020) and the dry season (December 2020). The Bankeng village (about 400 inhabitants^37^) is located at the transition from forest to savannah on the National Road N°1 linking Yaoundé to Kousséri (Fig. 1).

The locality is characterized by a classic equatorial rainfall regime with two dry seasons separated by two rainy seasons of unequal importance (from mid-March to June the long rainy season with important rainfall and from September to mid-November the short rainy season with less rainfall and more specifically from the first to the fifteenth of November when there is no more rain). The only malaria vector find in Bankeng belong to the genus of *Anopheles gambiae* complex, especially to *An. gambiae ss* (98.5%), and this vector exhibited a high level of resistance to almost all insecticides (93.9%) except to organophosphates^38,39^. As is the case across the country, the main control strategy used against exposure to mosquito bites is the Long-lasting insecticidal nets (LLIN) in Bankeng and the last distribution campaign which is universal coverage was taking place in 2019 during the survey period.The community had been sensitized a week earlier by community facilitators who invited them to participate in the study. After the study was presented to the community, all individuals who met the eligibility criteria and agreed to participate in the study were recruited after signing an informed consent form. For participants under the age of 21, parental or guardian consent and participant assent were obtained.

To limit the selection and information bias, participants were consecutively recruited during the two seasons and participation in the study was voluntary. Eligibility for inclusion was defined as local residents who had not traveled outside the study area in the previous 3 weeks, as it is known that antibodies to the salivary peptide drop significantly in 3 weeks when exposure to Anopheles bites ceases or significantly reduces^23,25^. Pregnant women and individuals with severe clinical signs of malaria as defined by WHO^40^, were excluded.

The study was approved by the National Ethics Committee for Human Health Research of Cameroon (CE N˚1840/CRERSHC/2019), and administrative authorization was obtained from the local head. Participation in the study was completely anonymous and without compensation.

### Entomological survey

*Anopheles* mosquito density and *Anopheles* species diversity were assessed during the two seasons. Adult mosquitoes were collected from the legs of 20 adult volunteers in 5 randomly selected houses in the village between 6 p.m. and 8 a.m. on two consecutive nights using the HLC method. Bankeng was divided into 5 sectors of around 20 houses each, and one house was randomly selected in each sector given the 5 selected houses.

For each house, HLCs of adult mosquitoes were performed both indoors (one collection point) and outdoors (one collection point) in houses at least 50 m apart. For both surveys (rainy and dry-seasons), the same houses were used for mosquito’s collection. Two volunteers worked during the first half of the night (from 6 p.m. to 0 a.m.), and the other two volunteers worked during the second half of the night (from 0 to 8 a.m.). Volunteer collectors were rotated between collection sites on different collection nights to minimize sampling bias. After mosquito capture, each collection tube containing mosquitoes was transferred to labeled bags according to the time of capture for assessment of mosquito aggressiveness by measuring the HBR. Collected adult *Anopheles* were then morphologically identified to species according to the dichotomous keys of Gillies and Coetzee^41^. HBR was expressed as the number of female *An. gambiae s.l.* per person per night, averaged for both outdoor and indoor collection sites, and averaged for both the rainy and dry-seasons.

Each mosquito was then preserved in 1.5 ml Eppendorf tubes containing silica gel and stored at − 20 °C until further analysis. The head-thorax-midgut was used for serologic analysis to identify the infected mosquito and the leg wing for molecular analysis to identify *Anopheles* subspecies.

For molecular identification, DNA was extracted from the legs and wings of each specimen according to the protocol of Collins et *al.*^42^ and PCR amplification for *Anopheles* subspecies identification was performed following the method of Fanello et al. which identifies species of *An. gambiae* complex^43^.

For the estimation of mosquito IR, the enzyme-linked immunosorbent assay (ELISA) was used to detect circumsporozoite protein (CSP) antigen, and the IR was then calculated as the proportion of *Anopheles* species that tested positive for *Plasmodium falciparum* CSP antigen by ELISA. The head and thoracic-migut parts of each collected female *Anopheles* were separated from the rest of the body, homogenized in grinding buffer (0.5% casein, 0.1N NaOH, 1 × PBS) and tested for the presence of *P. falciparum* CSP Antigen^44^. To minimize false-positive CSP ELISA results, only high absorbance values (mean plus three standard deviations of negative controls) were considered. Once the IR was determined, the EIR was calculated for each season as the product of the IR and the HBR.

### Blood sampling and parasitological survey

For each period, the parasitological survey was preceded by the entomological survey 2 weeks before.

A drop of blood was taken from the fingertips of each individual and used for: (1) malaria rapid diagnostic test (SD Bioline Malaria Ag *P.f*/Pan Ag) performed directly on the field, (2) thick/thin blood smear realized on the field and examined at the laboratory at Centre Pasteur du Cameroun (CPC) for microscopic diagnosis and (3) dried blood spots on filter paper (Whatman 3 M) for molecular diagnosis of *Plasmodium* species and for the quantification of the level of human anti-gSG6-P1 IgG antibody response at the laboratory.

*Plasmodium falciparum* parasitaemia was determined by microscopic examination of Giemsa-stained thick-blood smears and read with mineral oil at 100X. Parasite density was determined as the number of parasites per microL and counted against at least 200 leukocytes r, by assuming a leukocyte density of 8000 cells/microL of blood^45^. Positive samples from the rapid diagnostic test (RDT) and microscopic specimens were confirmed by multiplex polymerase chain reaction (PCR) according to a previously described protocol that allows the identification of five species of *Plasmodium*: *P. falciparum*, *P. malariae*, *P. ovale*, *P. vivax* and *P. knowlesi*^46,47^.

During the parasitological survey, data collection sheets were used to collect data on socio-demographic characteristics (age, sex), GPS geographic location of home, and malaria prevention interventions (ownership and use of long-lasting insecticide-treated nets-LLITNs).

All individuals who tested positive for malaria infection using RDT were treated directly free of charge with artemether/lumefantrine combination therapy in accordance with the treatment guidelines of the Cameroon National Malaria Control Program. All volunteers for the entomological study and before the experiments also received antimalarial chemoprophylaxis consisting of 100 mg doxycycline per day for the duration of the study and for one month afterwards.

### gSG6-P1 antigen and Human IgG antibody responses

The recombinant *Anopheles*-specific salivary peptide gSG6-P1 peptide previously described by Poinsignon^23^, was designed, synthesized and purified (> 95%) by Genepep SA (Montpellier, France). All peptide batches were delivered in lyophilized peptide form, then suspended in ultrapure water and stored in aliquots at − 20 °C until use.

The level of IgG Ab response to gSG6-P1 salivary peptide was measured on whole blood elutes obtained from standardized dried blood spots according to a protocol previously described by Drame et al*.*^48^. Briefly, 96-well Maxisorp plates (Nunc, Roskilde, Denmark) were precoated with 100 microL/well of gSG6-P1 salivary peptide solution (20 μg/ml of antigen in 100 microL of PBS 1 m, pH 7.4) and incubated for 2h30min at 37 °C. The plates were then blocked with 300 microL/well of protein-free PBS blocking buffer, pH 7.4 (Pierce, Thermo Scientific, France) for 45 min at 37 °C. Each eluted blood sample (1/50 dilution in PBS-Tween 1%) was incubated overnight at 4 °C in triplicate in the microtiter plates (2 wells with antigen labeled “Ag^+^” and 1 well without antigen labeled “Ag^-^”). A volume of 100 microL/well of biotin mouse anti-human IgG (secondary Ab solution, BD Pharmingen™) diluted at 1/2000 in PBS-Tween 1% buffer was added after 1 h 30 min of incubation at 37 °C. Streptavidine-peroxydase conjugate (GE Healthcare UK) was then added at 1/2000 dilution and incubated at 37 °C for for 1 h at 100 microL/well. Finally, a volume of 100 microL/well of substrate ABTS (2,2-azino-bis (3-ethylbenzthiazoline-6-sulfonic acid) diammonium; Thermo Scientific) in buffer solution (0.05 M citrate buffer, pH 4) and containing 10 microL of oxygenated water (30%) was added and incubated for 2 h in the dark at room temperature for the colorimetric development and the optical density (OD) was read at 405 nm.

IgG response to the salivary peptide was also measured in 34 non-*Anopheles* exposed individuals from France (European volunteer blood donors with no history of travel to malaria endemic countries) and were used as negative controls. Blood samples for exposed individuals (sample of patient tested positive to malaria and also as they living in endemic area of malaria was obtain from the Haematology service of Centre Pasteur du Cameroun) were used as positive control. All these control were used to control plate-to-plate variation and the validation of our experiment.

### Data and statistical analysis

The IgG response to gSG6-P1 antigen was measured at the individual level and expressed as the ΔOD value: ΔOD = ODx − ODn, where ODx is the mean of the individual OD in both antigen wells and ODn is the individual OD in the well without gSG6-P1 antigen (to remove any non-specific reaction and background in each sample). For each sample analyzed, the experiment was validated only if the coefficient of variation (CV) between the two Ag^+^ wells was < 20%. Samples with CV > 20% were reanalyzed. All subjects with ∆OD > 0.20 (cut-off) were defined as immune responders to the gSG6-P1 peptide. The cut-off value was defined as the mean ΔOD of the negative control plus three times the standard deviation (SD) and the mean ∆OD of the positive control was higher to 0.20 (∆OD > 0.20).

The Fisher exact test was used to compare qualitative variables. After verifying that the specific IgG response data (expressed as ΔOD) did not assume a Gaussian distribution, the non-parametric Mann–Whitney U test was used to compare antibody levels between two independent groups, the Wilcoxon matched-pairs test was used for the comparison of two paired groups and the Kruskal–Wallis for multiples comparison test between more than two groups. Only values p < 0.05 were considered significant. All statistical analyses were performed with GraphPad Prism5 software (San Diego, CA, USA).

### Ethical approval and consent to participate

This study was conducted according to the ethical guidelines for human research in Cameroon. The protocol of the study was approved by the Regional Center Ethics Committee for Human Health Research (CE N ˚ 1840/CRERSHC/2019). Prior to their enrollment in the study, the population was first informed on the purpose and process of the investigation (background, goals, methodology, study constraints, data confidentiality, and rights to opt out from the study). Signed informed consent was obtained from all participants above 21 years of age or older who agreed to participate in the study, and from the parent or legal guardian of minors (under 21 years of age in Cameroon). For minors (11–20 years of age), a signed informed assent was also obtained in accordance with the Declaration of Helsinki. Participation was voluntary, anonymous, and without compensation.

## Results

### Socio-demographic characteristics, LLITNs use and prevalence of malaria infection

General characteristics and prevalence of malaria infection in the population survey are presented by season (Table 1). A total of 190 individuals (107 in the rainy season and 83 in the dry season) were included in the study. The majority of participants were male regardless of season, 52.3% in the rainy season and 51.8% in the dry season. The mean age of the population was higher in the dry season (25.76 ± 22.23) compared to the rainy season (23.9 ± 23.5), but this difference was not statistically significant.

**Table 1:** Basic characteristics of population survey.

Parameter	Rainy season (%) 107 (56.3)	Dry season (%) 83 (43.7)	p-value
Gender			
Males	56 (52.3)	43 (51.8)	1
Females	51 (47.7)	40 (48.2)	
Mean age (SD), years	23.9 (23.5)	25.7 (22.2)	0.3321
Reported slept under LLITNs			
Yes	71 (66.3)	63 (75.9)	0.1992
No	36 (34.6)	20 (24.1)	
RDT			
Positive	60 (56.1)	62 (74.7)	0.0095
Negative	47 (43.9)	21 (25.3)	
Microscopy			
Positive	68 (63.5)	48 (57.8)	0.4554*
Mean GP, p/microL [95%CI]	1214 [804.6–1830]	856.1 [494.1–1483]	0.5719
Negative	39 (36.5)	35 (42.2)	–
Multiplex PCR			
Positive	83 (77.6)	51 (61.4)	0.0397*
* P. falciparum*	81 (97.7)	33 (64.7)	˂ 0.0001
* P. malariae*	0 (0.0)	2 (3.9)	
* P. ovale*	0 (0.0)	7 (13.7)	
* Pf/Pm*	1 (1.2)	3 (5.9)	
* Pf/Po*	1 (1.2)	5 (9.8)	
* Pm/Po*	0 (0.0)	1 (1.9)	
Negative	24 (22.4)	32 (38.7)	–
Over all infected (PCR)			
< 5 years	18 (16.82)	11 (13.25)	Reference
[5–16 years]	24 (22.49)	18 (21.68)	0.8070
[16–60 years]	31 (28.97)	18 (21.68)	1.000
≥ 60 years	8 (7.47)	4 (4.82)	1.000
Immune responder to gSG6-P1	106 (99.07)	79 (95.18)	0.1696

Data are number and proportion (%); unless otherwise indicated; SD: Standard deviation; LLITNs, Long Lasting Insecticide-Treated Nets; Mena GP, mean geometric parasitemia; Pf/Pm: coinfection of *P. falciparum* with *P. malariae*; Pf/Po: coinfection of *P. falciparum* with *P. ovale*; Pm/Po: coinfection of *P. malariae* with *P. ovale*; P-value show the statistical significant or not between rainy and dry season; *Comparison between positive and negative group.

More than 60% of the participants in both seasons reported sleeping under a bed net every night, more in the dry season (75.9%) than in the rainy season (66.3%).

As observed for malaria diagnosis by microscopy and PCR, the prevalence of malaria infection was higher in the rainy season (63.5% by microscopy and 77.6% by PCR) compared to the dry season (57.8% by microscopy and 61.4% by PCR). *P. falciparum (Pf)* infection remains the most prevalent of the parasites detected with 97.6% and 64.7% in the rainy and dry seasons, respectively, and this difference was not statistically significant (p ˂ 0.0001).

Regarding the other species found *P. malariae* (*Pm*) and *P. ovale* (*Po*), mono-infections were found only in samples collected during the dry season. The two cases of co-infection (*Pf*/*Pm* and *Pf*/*Po*) were found in samples collected during the both seasons. The distribution of species differed significantly between seasons (p ˂ 0.0001).

Among the 107 individuals included in rainy season, 31 of them were present in the dry season. Female were the most represented 18 (58.06%), the mean age was 28.46 ± 24.55 vs 28.99 ± 24.09 in rainy and dry season respectively. The malaria prevalence among them was higher in the dry season (83.87%) compared to the rainy season (54.83%) and the difference was significant (p = 0.0262).

### Specific IgG response level to An. gambiae gSG6-P1 salivary peptide according to the season

**Figure 2:**
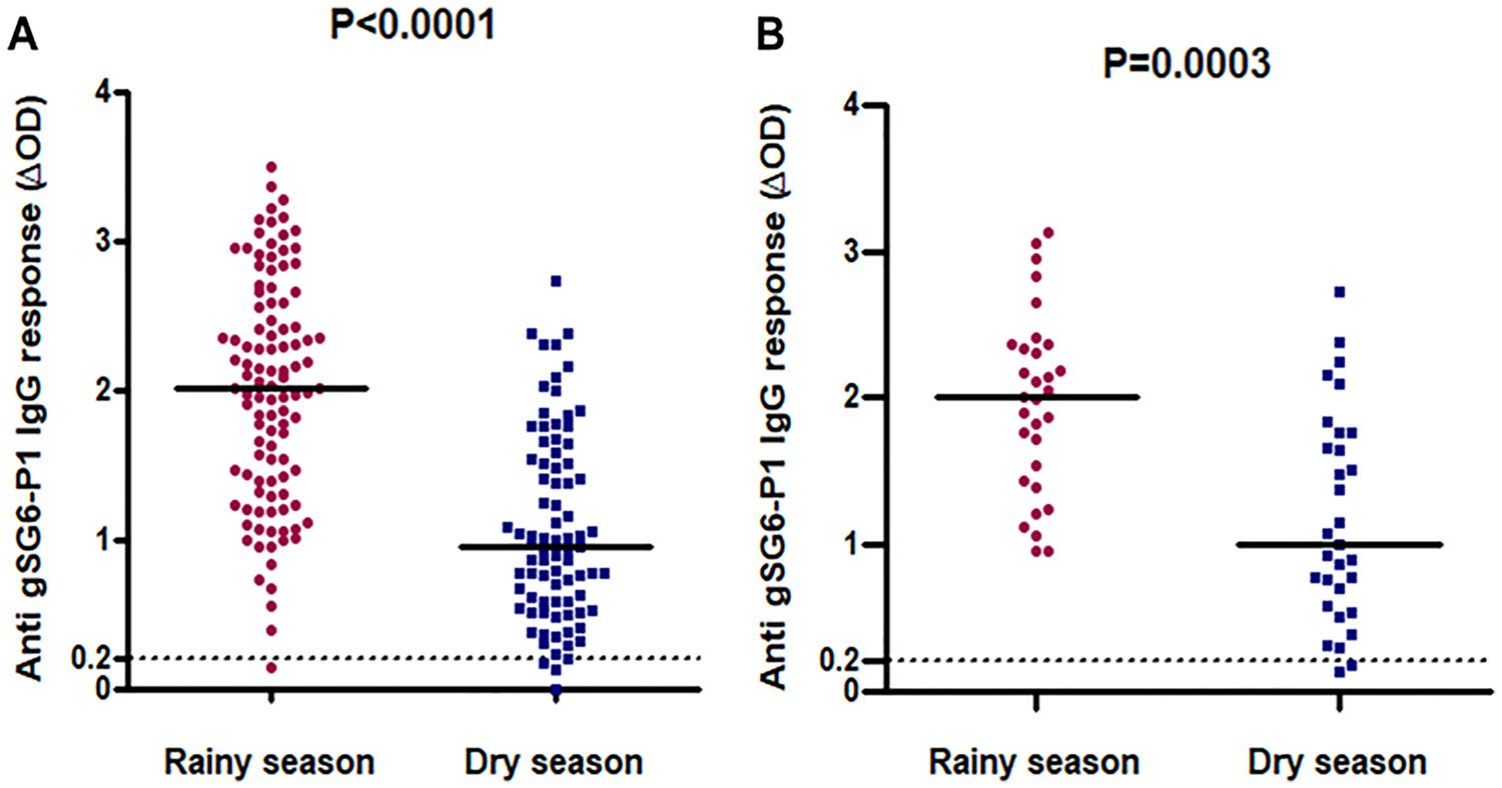
IgG response levels to An. gambiae gSG6 P1 salivary peptide in human population in dry- and rainy-season in whole population included in each season (**A**) and among the 31 individuals following in dry- and rainy-season (**B**). Dot plots indicate the individual specific IgG level (ΔOD value) for samples collected in rainy- and dry-season, and bars represent median value in each group. The black dotted line represents the cut-off of IgG response. Statistical significance between the seasons is indicated (non-parametric Mann–Whitney U-test that compare median for all the population; (**A**) and, the Wilcoxon matched-pairs test used to compare median of the follower groups; (**B**)).

The levels of specific IgG Ab responses to the gSG6-P1 salivary peptide in the human population of Bankeng were assessed during the rainy and dry seasons and, significant variations were observed between the two seasons (Fig. 2).

Despite of the inter-individual heterogeneity in the two periods, the median of the specific IgGlevels differed significantly (p ˂ 0.0001) and was higher in the rainy season (median ΔOD: 2.012, 95% CI 1.849–2.137) than in the dry season (median ΔOD: 0.965, 95% CI 0.953–1.241) (Fig. 2A). Furthermore, this trend of high specific IgG levels is also observed in the rainy season when we compare the IgGresponse levels of the 31 individuals included in the two periods (Fig. 2B).

IgG responses decreased significantly in the dry season (median ΔOD: 0.998, 95% CI 0.917–1.438) compared to the rainy season (median ΔOD: 2.011, 95% CI 1.745–2.195) among these followed individuals (p ˂ 0.0001). The rainy season can be classified as a high anti-gSG6-P1 IgG response and the dry season as a low anti-gSG6-P1 IgG response using the median IgG level or the percentage of immune responders that occurred.

The level of Anti-gSG6-P1 IgG response was also analysed according to age group, sex and the use of LLINs among the population of Bankeng in each season.

Age was stratified in four groups as follow: ˂ 5 years, [5–16] years, [16–60] years and ˃ 60 years in the general population and also in the follower individuals. The level of anti-gSG6-P1 IgG response according to age group did not vary significantly with respect to rainy (p = 0.9262) and dry season (p = 0.4104). However, we observed a higher level of anti-gSG6-P1 IgG response in the age group of [5–16] years (median ΔOD: 1.703, 95% CI 1.197–1.955) compared to the group of [16–60] years (median ΔOD: 0.6403, 95% CI 0.4115–1.1690) and this difference was statistically significant (p = 0.0041). The same trend was observed in the rainy season, but without significant difference (p = 0.3666) (Supplementary data S1).

Females and males exhibited approximately the same level of IgG response to *An. gambiae* gSG6-P1 salivary peptide independently of the season and this difference was not statistically significant in the rainy season (p = 0.7528) and in the dry season (p = 0.7053) (Supplementary data S2). The same trend was observed in the follower-up individuals in the rainy season (p = 0.6742) and in the dry season (p = 0.921).

According to the use of mosquito nets and the level of IgG response to *An. gambiae* gSG6-P1 salivary peptide, analyses revealed in overall population no significantly difference observed according to the use of LLINs independently the season (Supplementary data S3). No difference was observed between individuals that reported sleeping under LLINs and those that did not in each season. The level of IgG anti-gSG6-P1 salivary peptide response seemed to be equal among people who reported using LLINs frequently (median ΔOD: 2.052, 95% CI 1.805–2.164) and those who did not use LLINs (median ΔOD: 1.968, 95% CI 1.754–2.265) in both rainy with the same trend in dry (p ˃ 0.05). In the follower group the level of IgG response was lower in people that use frequently LLINs (median ΔOD: 0.8981, 95% CI 08.685–1.507) than in people that did not use LLINs (median ΔOD: 1.231, 95% CI 0.5907–1.709) in the dry season, but the difference was not significant (p = 0.9820). The same observation was made in the rainy season (p = 0.7017).

### IgG levels to the An. gambiae gSG6-P1 salivary peptide according to malaria infectious status

**Figure 3:**
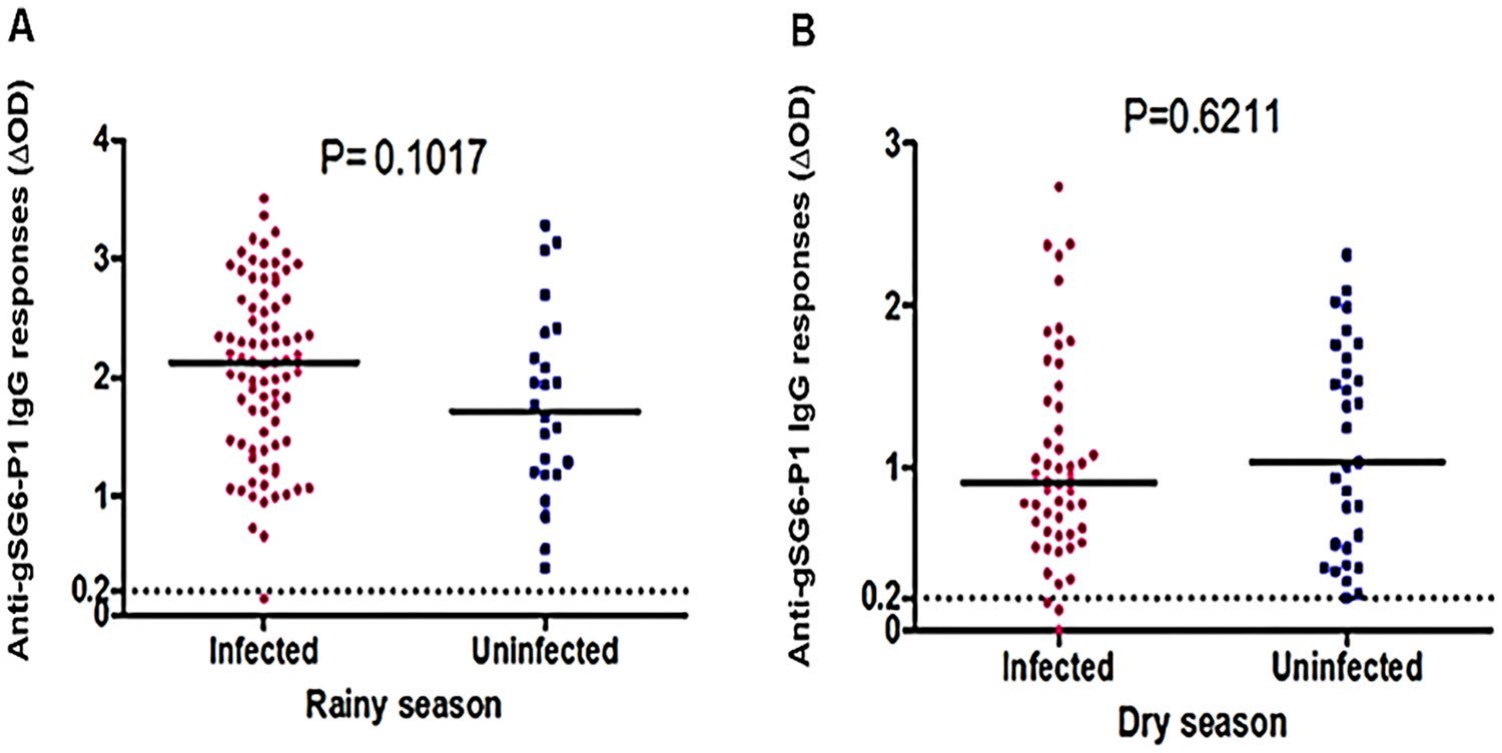
IgG response to *An. gambiae* gSG6 P1 salivary peptide according to malaria infection in dry-season (**A**) and rainy-season (**B**). Dot plots indicate the individual specific IgG level (ΔOD value) for *Plasmodium* infected and uninfected individuals, and bars represent the median value in each group. The black dotted line represents the cut-off of the IgG response. Statistical significance between the groups of individuals in each season (dry or rainy season) is indicated (non-parametric Mann–Whitney U test).

Specific IgG levels to the gSG6-P1 peptide were evaluated according to the malaria infection status studied and presented for the rainy season (Fig. 3A) and dry season (Fig. 3B).

The data showed variation within and between *Plamsodium* infected and non-infected individuals depending on the season. We observed the inter-individual heterogeneity of specific IgG levels to gSG6-P1 peptide, the median of specific IgG levels in *Plamsodium* infected and non-infected individuals did not differ significantly whatever the season. As observed, the median of anti-gSG6-P1 IgG levels appeared to be higher in *Plamsodium* infected than in non-infected samples collected during the rainy season, but this difference was not statistically significant (p = 0.1017). Using parasite densities groups (1–100; 101–5000 and above 5000 parasites per microL), no statistically significant difference (p ˃ 0.05) was observed (Supplementary data S4) regardless of the season. It should be noted that some relatively high specific IgG responses were also observed in uninfected samples (Fig. 3B). Data were also analysed with the follower group (the 31 individuals present during the two sampling periods (rainy and dry season)). The same trend was observed when compared the level of the IgG response with the infected and uninfected individuals (Supplementary data S5).

### Level of IgG response to the gSG6-P1 salivary peptide according to entomological parameters and season of transmission

A total of 334 anopheline specimens were collected (304 in the rainy season and 30 in the dry season). Those collected in the rainy season belonging to 3 species *An. gambiae s.l.* 301 (99.0%), *An. funestus* 01 (0.3%) and *An. nili* 02 (0.7%) and only the genus of *An. gambiae s.l was collected* in the dry season, showing a higher density of *Anopheles* in the rainy season. Among the 301 *An. gambiae s.l collected* in rainy season, 300 (99.67%) belonging to the sub-genus of *An. gambiae s.s* and 01 (0.33%) to *An. coluzzi*, identified by PRC. The entomological data indicated that mosquitoes had bitten both inside and outside the houses. The intensity of exposure to *An. gambiae s.l*. bites (the most abundant: 99.0% collected in the rainy season and 100.0% in the dry season) differed globally and also varied according to the season. Mosquitoes were more aggressive in the rainy season (HBR = 15.05 b/p/n) than in the dry season (HBR = 1.5 b/p/n).

Overall, 32 of the 301 *An. gambiae s.l.* collected in the rainy season were positive for *P. falciparum* CSP by ELISA, giving a mosquito IR of 10.63% and a higher risk of malaria transmission with an EIR of 1.42 ib/p/n. None of the mosquitoes collected during the dry season were positive for CSP (Table 2).

**Table 2:** *An. gambiae s.l.* mosquito’s biting behaviour and malaria transmission risk in Bankeng.

	n persons collecting (20)	Rainy season				Dry season			
		n mosquito (304)	HBR (b/p/n)	SI (%)	EIR (ib/p/n)	n mosquitos (30)	HBR (b/p/n)	SI (%)	EIR (ib/p/n)
Outdoor	10	147	14.7	10.63	1.42	16	1.6	0.0	0.0
Indoor	10	154	15.4			14	1.4		

n: number of.

**Figure 4:**
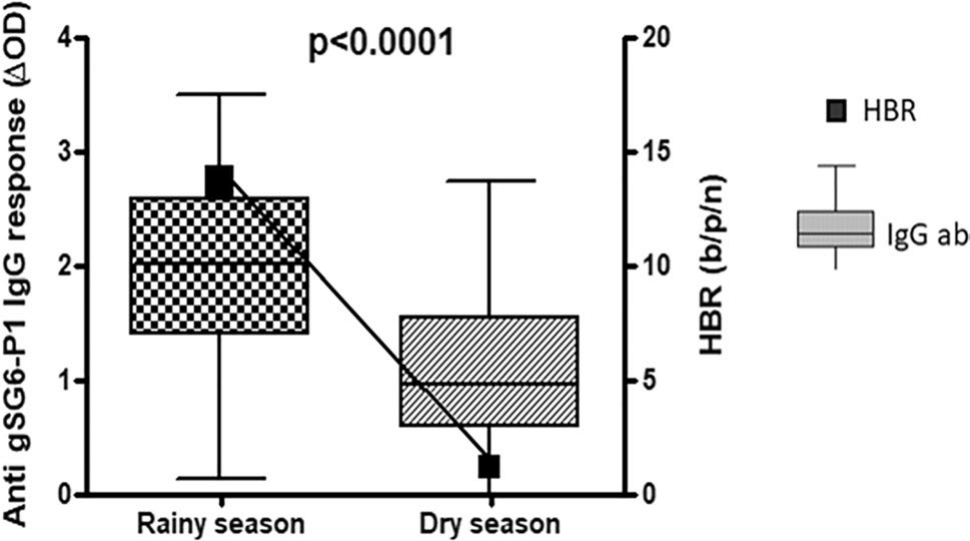
IgG response to *An. gambiae* gSG6 P1 salivary peptide according to the malaria vector aggressiveness in Bankeng. Boxes represent the individual specific IgG levels (ΔOD value) and black squares represent seasonal mosquito aggressiveness, i.e. human biting rate (HBR). Statistical significance between seasonal mosquito aggressiveness (dry season and rainy season) is indicated (non-parametric Mann–Whitney U-test).

The level of IgG response to the gSG6-P1 salivary peptide could be associated with the level of human exposure to malaria vector bites in Bankeng. We analyzed the level of this immune response according to HBR estimated in both rainy and dry seasons (Fig. 4). The median value of the specific IgG response level to the gSG6-P1 peptide was significantly higher in the rainy season, which correlated with higher HBR compared to the low HBR found in the dry season. This shows that the level of specific response to the gSG6-P1 peptide is associated with the density of malaria vectors and consequently with the level of exposure to the bite of these mosquitoes.

## Discussion

Improving malaria surveillance in an area where the malaria infection or disease is prevalent using a simple, highly sensitive and accurate tool is essential. A complementary indicator that allow to assess the level of human exposure to *An. gambiae* bites in the context of reducing malaria by at least 90% by 2030 as targeted by the WHO is very important to validate worldwide^40^. The present study focused on the application of the Anopheles gSG6-P1 salivary biomarker to assess vector bite exposure and mosquito density in a rural area and according to transmission season (dry and rainy seasons).

Data from the study conducted in the village of Bankeng, Cameroon, in the Central African Region, have showed that *An. gambiae s.l.* represented 99% of the *Anopheles* mosquito’s collected during the entomological survey in both the dry and rainy seasons. A similar distribution was reported in a recent study in the same area, indicating that the population of the village is highly exposed to *An. gambiae*^39^.

The level of IgG response to the gSG6-P1 salivary peptide correlated significantly with mosquito density. In fact, the level of IgG response, as well as the aggressiveness *Anopheles*, was higher in the rainy than in the dry season. This is in line with numerous previous studies that have reported this specific immune response as a relevant tool to assess mosquito density according to the areas in the West African region and in Asia^23,24,31,49^. These data demonstrated the relevance of using this biomarker to distinguish seasonal variations in human exposure to *Anopheles* bites and mosquito density.

Infected mosquitoes were found only during the rainy season. The sporozoite IRs were higher (10.63%) than what is generally reported as 5% of field mosquitoes being infected^50,51^, collected by HLC, and consequently the EIR was 1.42 ib/p/n with infected mosquitoes found in the rainy season. No infected mosquitoes were found in the dry season, probably due to the low density of mosquitoes collected, which may indicate the potential limitations of entomological methods in the dry season. Our data suggest that people in Bankeng are at high risk of being bitten by infected mosquitoes during the rainy season, and therefore at high risk of malaria transmission during the same period.

Malaria transmission is generally thought to be higher during the rainy season, when environmental conditions are favorable for the development of Anopheles larvae, than during the dry season, when mosquitoes do not breed. As expected, our results show that the prevalence of malaria infection was higher in the rainy season compared to the dry season. In the same line, entomological data clearly indicated a higher HBR with *Anopheles* infective bites in the rainy season, but not with infective bites in the dry season, even when individuals were also found infected with *Plasmodium* parasites.

Indeed, parasitological data indicated that prevalence and *Plasmodium* densities were not similar between the rainy and dry seasons, which seems to confirm the immunological observations when comparing globally infected individuals in the rainy and dry seasons. The malaria infection status (infected and uninfected) reported in the rainy season and compared with that observed in the dry season reinforces the immunological observations, indicating that individuals, whether infected or not, were exposed to the same level of vector bites.

The level of IgG anti-gSG6-P1 was higher in infected individuals compared to uninfected individuals in the rainy season with no significant difference, in contrast to the dry season where uninfected individuals appear to have a high level of Ab response. However, a significant difference between infected and uninfected individuals has been reported, with higher levels of IgG anti-gSG6-p1 in infected individuals^29,30,32,33,35^. On the contrary, we did not make the same observation and our results are comparable to those obtained by Traore et *al.* in Ivory Coast according to the rainy or the dry season^26,27^. This trend remains the same when we compared the level of the IgG Ab response between only the 31 individuals present during the two sampling periods (rainy and dry season). Although this sample size is small and may be biased, it remains that when there is high exposure to *Anopheles* bites, the level of response between infected and uninfected individuals may not be significantly different^26^. This observation suggests that IgG anti-gSG6-p1 is not a necessary a biomarker of infection for the higher level of Ab response of infected individuals in the rainy season, period of higher transmission, although it is a good biomarker of exposure to mosquito bites and vector densities according to season^26,27^ and to the study site^26,35^. Interestingly, infected individuals were found in the dry season, but no infected mosquitoes were found. These observations show the limitations of entomology, as the density of mosquitoes in the dry season is very low to detect infective bites and can lead to confusion. However, regardless of the aggressiveness of the mosquitoes (low HBR), individuals are still exposed to the bites of a few circulating Anopheles. It cannot be excluded that the observed prevalence of *Plasmodium* infection is a case of recrudescence of submicroscopic parasitemia, which increases over time and is detected during periods of low transmission.

No association with the level of IgG response to the gSG6-P1 antigen according to the gender was observed in the population study. Indeed, the IgG response to gSG6-P1 peptide could not differentiate human exposure to *Anopheles* bites regardless of the gender. Making this biomarker a good tool that can be used in all the population independently of the sex as reported in our previous study^35,36^ and also in others studies in West and East Africa^32,33^. The level of the immune response did not vary with regard to age group and this was also observed in our previous study^36^_,_ other study reported no association with this immune response and the age according to season^27^. However, some studies reported an association between age and the level of IgG to gSG6-P1 antigen^27,30,48^, the IgG response increasing with age as people gradually acquired the immunity against *Anopheles* mosquito saliva.

The specific IgG response to the gSG6-P1 peptide has also been shown in several studies in Senegal, western Angola and Kenya to be a suitable immunological biomarker for assessing low levels of human exposure to *Anopheles* bites^24,33,34,52^, as it was the case in the dry season in the present study. We found an association between mosquito density and the level of immune response to the salivary peptide. This shows the importance of this biomarker even in the dry season when mosquito densities are low, because even though mosquito densities are low, people are being bitten. And even though it is lower than in the wet season, the biomarker still detects the immune response. All of these data tend to support the proposal that the IgG response to the salivary peptide appears to be an effective biomarker for detecting the reduction in exposure to *Anopheles* mosquito bites. In fact, this specific immune biomarker was able to detect the reduction in exposure to vector bites that may be due to vector control strategies implemented in the fight against malaria. In contrast to others studies^24,53,54^, we did not find any significant difference between the use of mosquito bed nets and the level of IgG response to the gSG6-P1 antigen. This observation was also made in our previous study conducted in rural area^35,36^. The raison behind can be a bias in the questionnaire, indeed the information was given on the use of bed nets by participants without a through observation, if people really ownership or use it. Also, the integrity of the LLINs was not checked during the survey. Moreover, *Anopheles* bite equally outdoor and indoor as observed with entomological data in this area. Finally the type of house, mainly built in still that allow mosquito to get in suggested that people are exposed to mosquito bite continually in our study site. These information’s are very important in the monitoring of human exposure to malaria vectors bites. Indeed, it has been shown that after the use of vector control measures such as insecticide-treated nets (ITNs), insecticide sprays, insect repellents and bed nets, the levels of IgG anti-gSG6-P1 decrease significantly^25,53,54^. All these data show that this IgGis very labile and decreases significantly when there is no exposure to *Anopheles* bite. The results confirm the ability of this marker to detect exposure in areas where mosquito density is very low (depending on the season). It could therefore be very useful in the current context where vector control strategies tend to reduce human exposure to Anopheles bites. Such a marker would therefore allow the effectiveness of these strategies to be monitored in areas where mosquito densities have decreased.

## Conclusion

This study highlights the usefulness of this biomarker (IgG anti-gSG6-P1) to detect individual exposure to Anopheles bites during periods of low mosquito exposure. The IgG anti-gSG6-P1 peptide discriminates season of exposure and mosquito density variation, showing that when entomological methods lack effectiveness, as observed in the dry season with very low Anopheles density and no infective transmission bite, the immune response to mosquito bites can be easily used to identify individuals exposed to the bites of malaria vector. These observations could be very important for decision making regardless of the vector control strategies implemented by the National Malaria Control Program in Cameroon and also in rural African contexts.

### Supplementary Information

Supplementary Information.

## Data Availability

All data underlying the findings and the conclusions are included within the manuscript.

## Abbreviations

An.: *Anopheles*
ΔOD: Delta optical density
ELISA: Enzyme-linked immunosorbent assays
EIR: Entomological Inoculation Rate
IgG: Immunoglobulin G
gSG6-P1: Gambiae salivary gland protein 6-peptide 1
HBR: Human biting rate
HLC: Human landing catches
IR: Infection rates
RDTs: Rapid diagnostic tests
SIR: Sporozoïtes index rate
WHO: World Health Organization
