# Supplementary material for: Utility of plasma anti-gSG6-P1 IgG levels in determining changes in Anopheles gambiae bite rates in a rural area of Cameroon
Source: Sci Rep. 2024 Jun 21;14:14294. doi: 10.1038/s41598-024-58337-8 (PMC11192751; doi:10.1038/s41598-024-58337-8)
Supplement: Supplementary file 1 — Supplementary Information. [file 41598_2024_58337_MOESM1_ESM.docx]

Supplementary data

**S1.** IgG response to *An. gambiae* gSG6 P1 salivary peptide according to age group in rainy-season (A) and dry-season (B) in the general population and among the follower individuals in rainy (C) and dry season (D).

Statistical significance between the age groups in each season (dry or rainy season) is indicated (non-parametric Kruskal-Wallis, Multiple Comparison test).


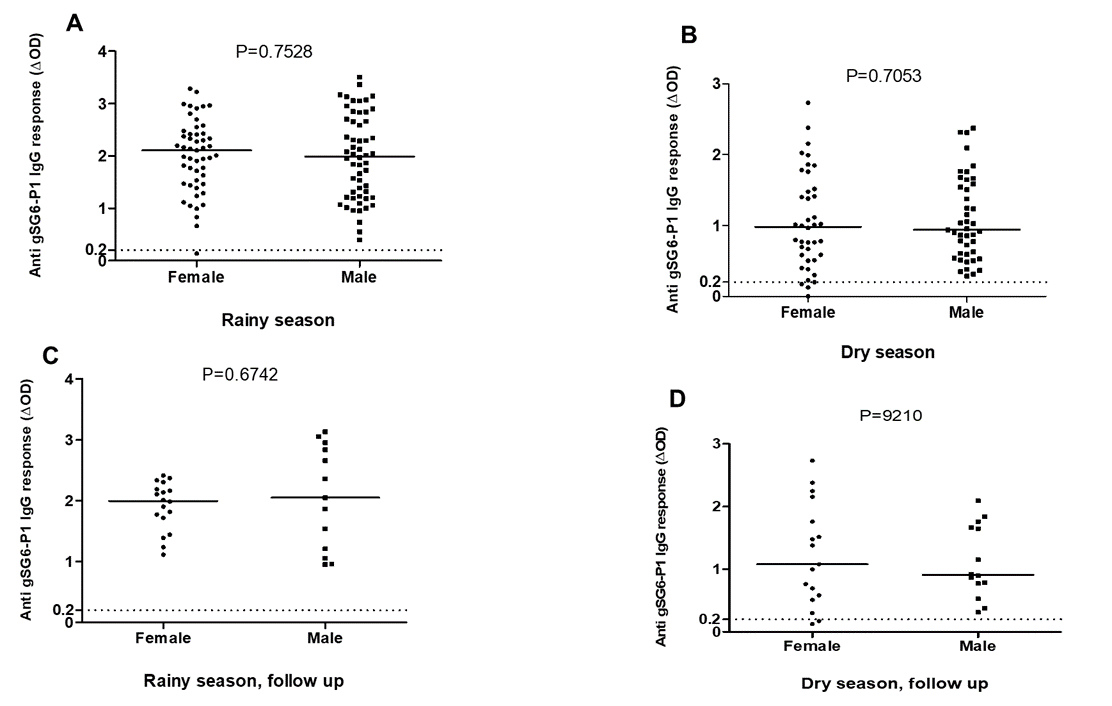


**S2.** Level IgG response to *An. gambiae* gSG6 P1 salivary peptide according to gender in rainy-season (A) and dry-season (B) in the general population and among the follower individuals in rainy (C) and dry season (D).

Statistical significance between gender in the population and the follower individuals in each season (dry or rainy season) is indicated (non-parametric Mann-Whitney U test).


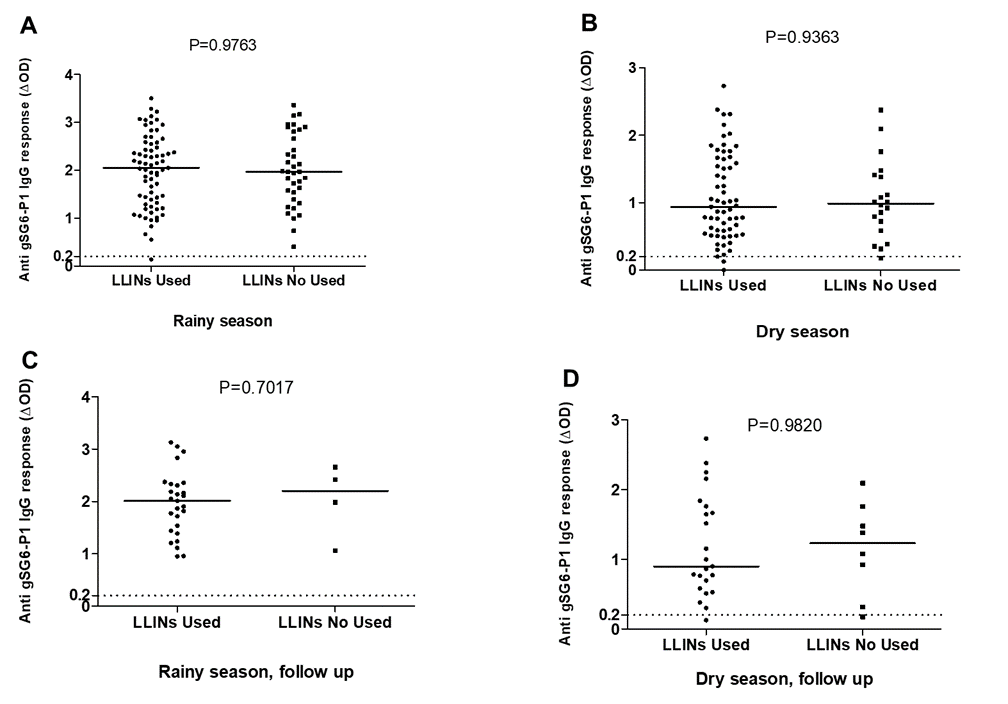


**S3.** The level of IgG response to *An. gambiae* gSG6 P1 salivary peptide according to the use of LLINs in rainy-season (A) and dry-season (B) in the general population and among the follower individuals in rainy (C) and dry season (D).

Statistical significance between peoples who use LLINs and peoples that did not among the population and the follower individuals in each season is indicated (non-parametric Mann-Whitney U test).

**S4.** The level of IgG response to *An. gambiae* gSG6 P1 salivary peptide according to the parasites density in rainy-season (A) and dry-season (B) in the general population

Statistical significance difference is indicated (non-parametric Kruskal-Wallis, Multiple Comparison test).

**S5.** The level of IgG response to *An. gambiae* gSG6 P1 salivary peptide according to the infected and uninfected in rainy-season (A) and dry-season (B) among the follower individuals.

Statistical significance between peoples infected and uninfected in each season is indicated (non-parametric Mann-Whitney U test).
